# Supplementary material for: Epidemiological model for the inhomogeneous spatial spreading of COVID-19 and other diseases
Source: PLoS One. 2021 Feb 19;16(2):e0246056. doi: 10.1371/journal.pone.0246056 (PMC7894958; doi:10.1371/journal.pone.0246056)
Supplement: S1 File — (PDF) [file pone.0246056.s001.pdf]

Supporting Information – Epidemiological model for the inhomogeneous spatial spreading of COVID-19 and other diseases

Yoav Tsori and Rony Granek

Description: In this Supporting Information we provide additional figures that are not provided in the main manuscript. Notations and symbols are identical to those used in the main text.

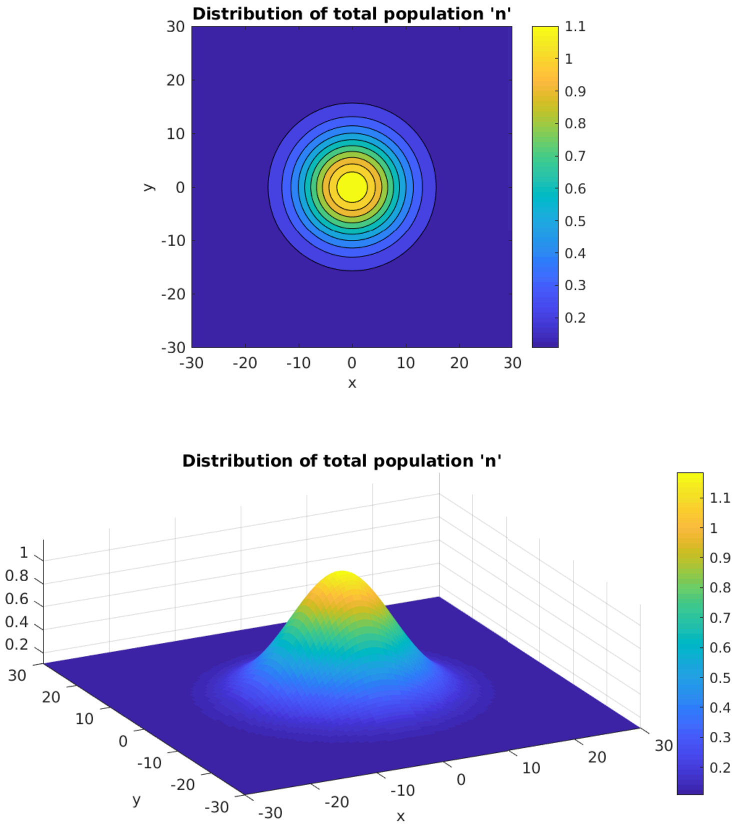

Fig. SI-1. Illustration of a city with Gaussian density. Distribution of the total population of a “city” $n(x, y)$ as given in Figs. 2, 4 and 5 of the main text. (a): a contour plot, (b): same information shown as a surface plot. The population density is $n(x, y) = 10ae^{-r^2/\ell^2} + a$, where $r^2 = x^2 + y^2$, $\ell = 10$, and a is chosen such that $(1/\text{area}) \int n(x, y)\text{d}x\text{d}y = 0.2$.

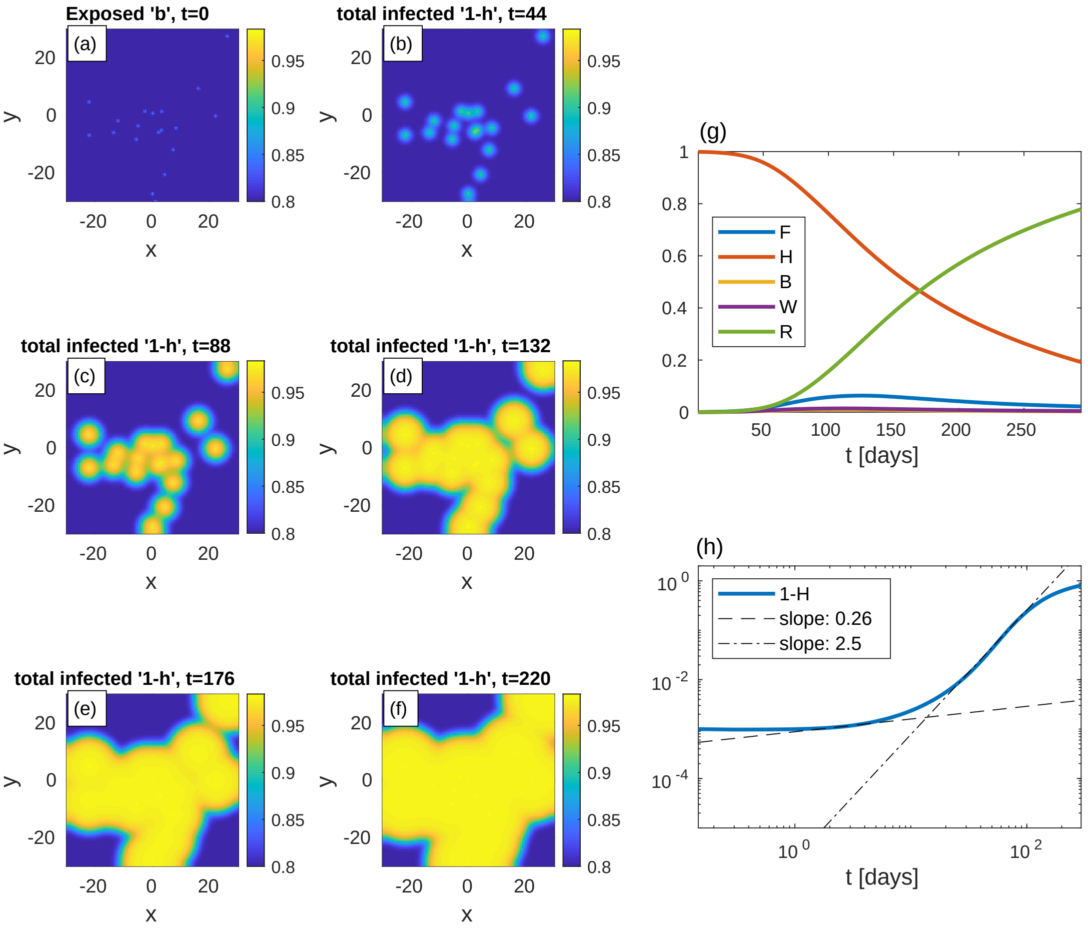

Fig. SI-2. Heat-maps for cumulative infections. Time evolution of an epidemic with randomly distributed infection centers. The simulation (numerical solution) is identical to that shown in Fig. 3 of the main text, but here the panels (a)-(f) show the *cumulative infected population* $b + w + f + r$, that is, $1 - h$. (a) At $t = 0$ (top-left panel), there are small infection centers of the latent population 'b' distributed randomly in space. $n = 0.2$ is uniform and all other populations are set initially to zero: $w = f = r = 0$. Panels (b)-(f) show the spread of the infected population '$1 - h$' as time progresses and (g)-(h) show the evolution of the total quantities.

Sensitivity analysis. It is instructive to know how sensitive our model is to the numerical choice of the parameters. Recall the definition of k and $\tilde{D}_k$ as $k = k_1 + zk_2$ and $\tilde{D}_k = k_2$, where k_1 is the infection rate within the same node, and k_2 is the infection rate between nearest-neighbor nodes and $z = 4$. In the main text we used the value $R_0 = 2.5$ for the basic reproductive number and $\tau_I = 16.6$ days yielding $k = 0.15$ days^{-1}. In order to perform sensitivity analysis we consider four different cases:

- Case 1: the same parameters as used in the main text:
  $k_1 = k_2 = 0.03$, yielding $k = 0.15$ and $\tilde{D}_k = 0.03$.

- Case 2: weaker nearest-neighbor infections than inter-node infections:
  $k_1 = 0.03$ and $k_2 = 0.01$. This yields $k = 0.07$ and $\tilde{D}_k = 0.01$.

- Case 3: smaller value of the basic reproductive rate: $R_0 = 1.5$, as applicable when people use protective measures such as face masks, etc. This value of R_0 leads to $k = 0.09$ and $\tilde{D}_k = 0.018$.

- Case 4: higher value of the basic reproductive rate: $R_0 = 4$. This value of R_0 leads to $k = 0.24$ and $\tilde{D}_k = 0.048$.

- Case 5: higher value of the basic reproductive rate: $R_0 = 5$. This value of R_0 leads to $k = 0.3$ and $\tilde{D}_k = 0.06$.

In all five cases above we used the same values of $\gamma_0 = 1/2$ days^{-1}, $\gamma_1 = 1/3$ days^{-1} and $\gamma_2 = 1/13.6$ days^{-1}, as in the main text.

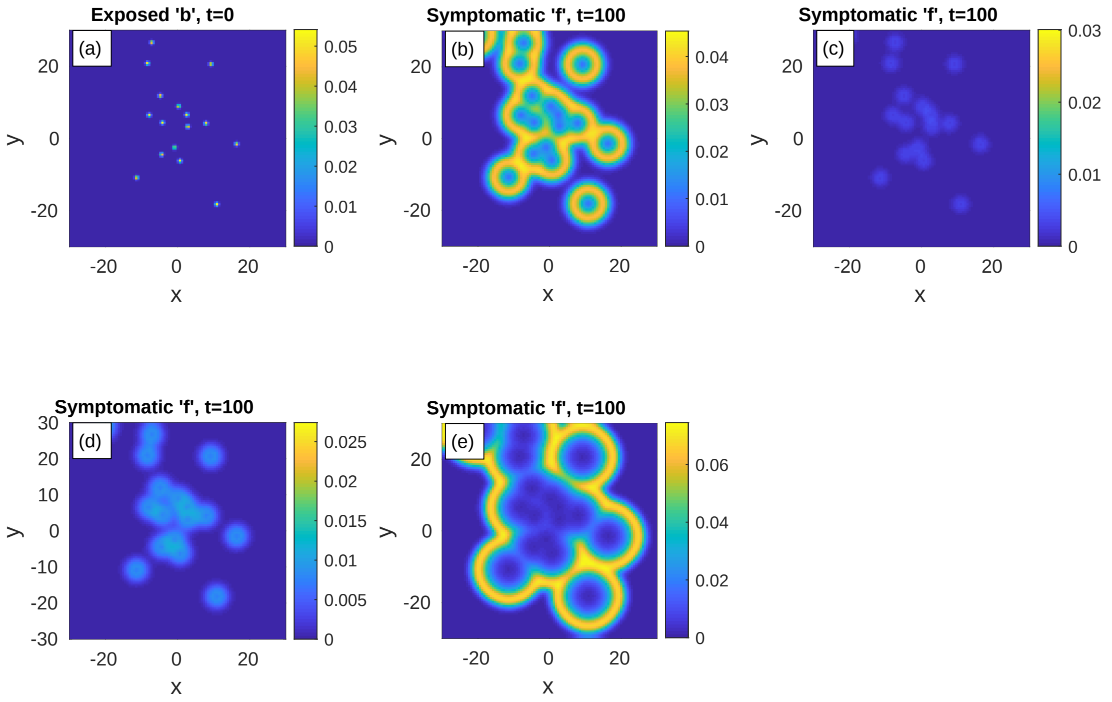

Fig. SI-3. Sensitivity analysis. Comparison of the time evolution of the epidemic in scenarios 1-4 described above. Panel (a) shows the shared initial conditions: at $t = 0$, there are small infection centers of the latent population 'b' distributed randomly in space. $n = 0.2$ is uniform and all other populations are set initially to zero: $w = f = r = 0$. All other panels show the resulting heatmaps of symptomatic population 'f' at the same time $t = 100$, according to (b): case 1, (c) case 2, (d) case 3, and (e) case 4. Clearly, when the basic reproductive rate is large, case 4, the epidemic spreads more quickly to uninfected regions. Case 3 is significantly slower due to the reduced value of R_0. Case 2 is the slowest due to the small value of inter-node infection rate k_2 and small diffusion-like coefficient $\tilde{D}_k$.

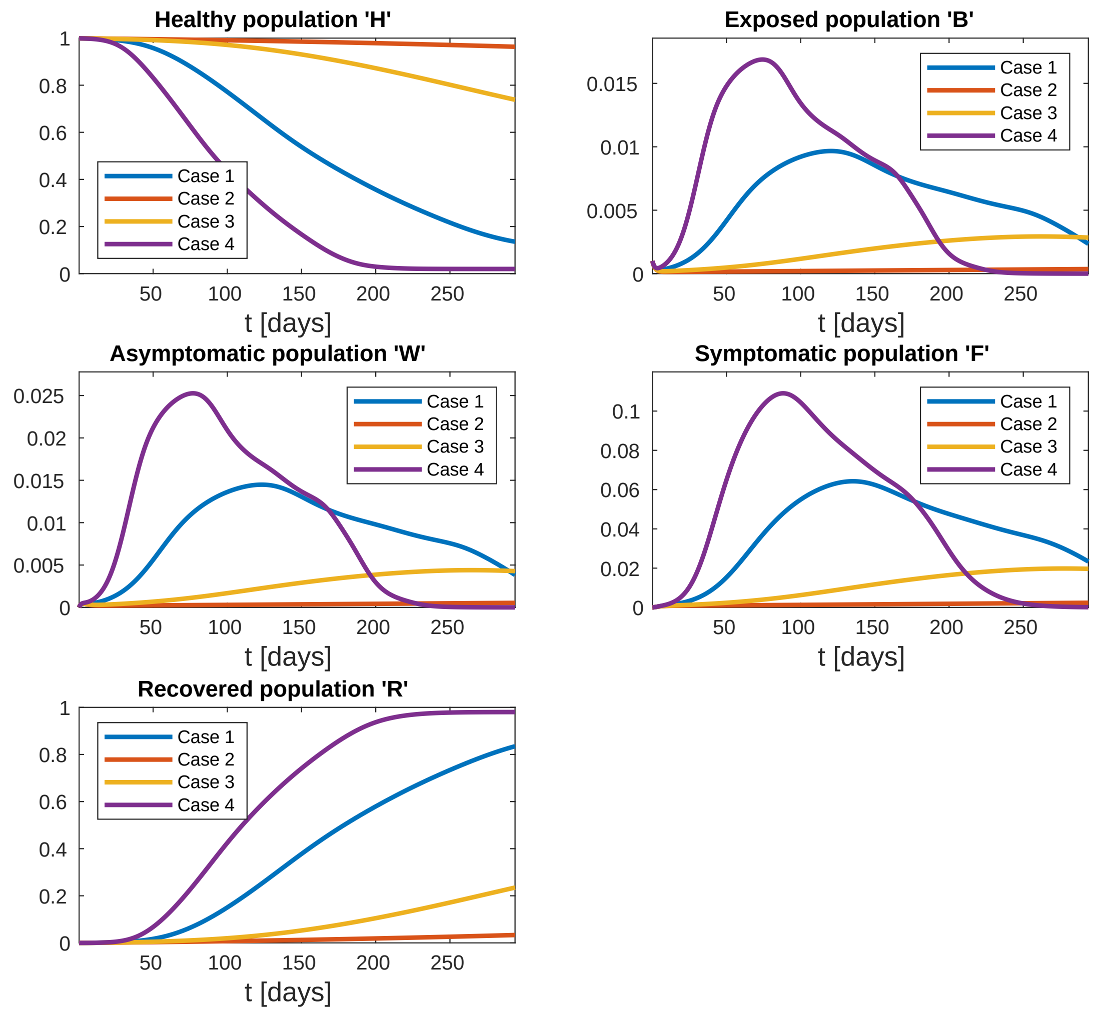

Fig. SI-4. Sensitivity analysis. Plots of the different sub-populations for each of the scenarios 1-4. Each panel shows the time evolution of a different sub-population integrated over the whole area, 'H', 'B', 'W'', 'F', and 'R'. Compared to the "baseline" scenario employed in the main text (case 1, blue curves), when the basic reproductive rate is large, case 4 (purple curves), the epidemic spreads more quickly resulting in more infected people and a smaller healthy population. Case 3 (yellow curves) is significantly slower due to the reduced value of R_0. Case 2 (red curves) is the slowest due to the small value of inter-node infection rate k_2 and small diffusion-like coefficient $\tilde{D}_k$.

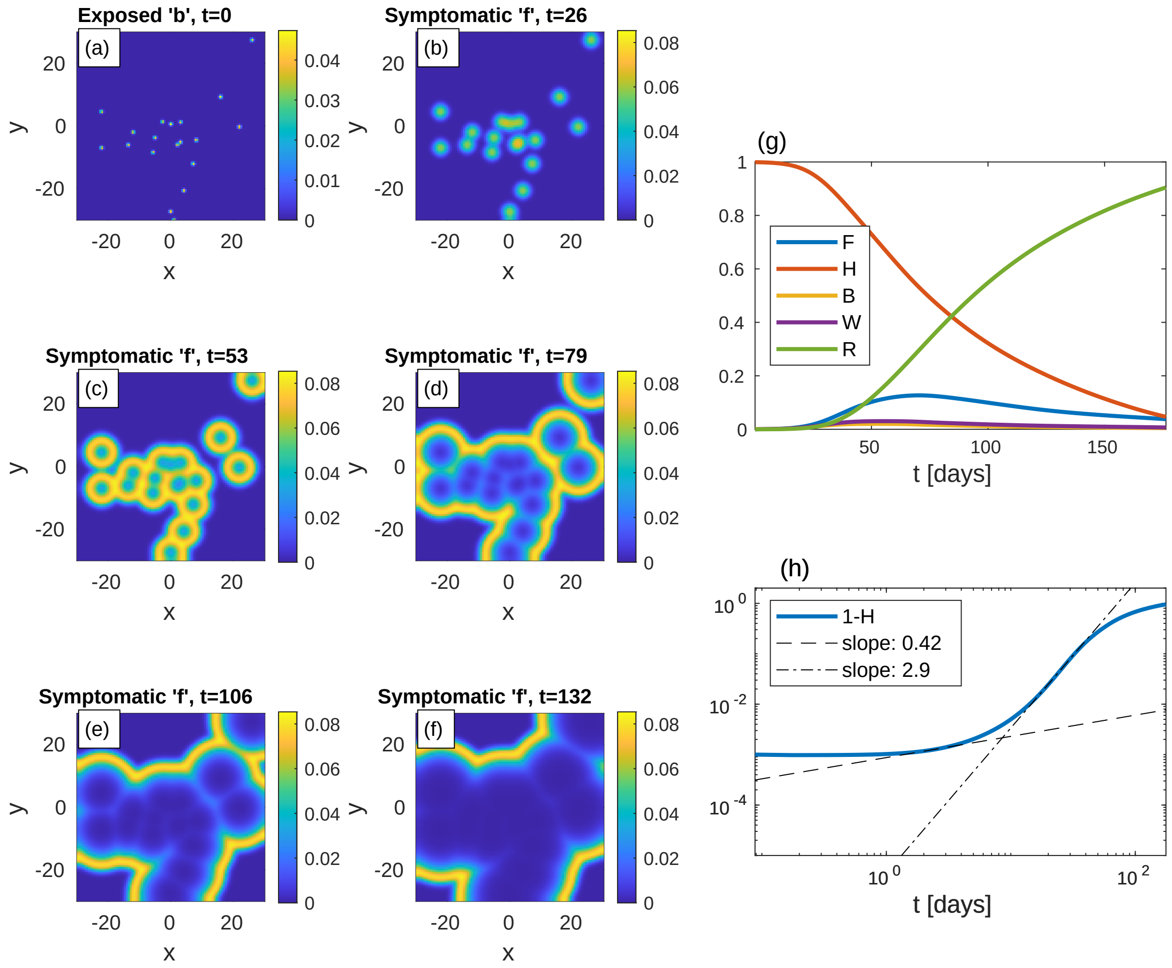

Fig. SI-5. Sensitivity analysis. Same initial conditions as in Fig. 4 of the main text but for (sensitivity analysis) scenario 5, i.e. a value of R_0 that is twice larger – $R_0 = 5$. The chosen times for the snapshots are shorter than in Fig. 4. The heat-map patterns shown here (though at shorter times) are almost indistinguishable from those appearing in Fig. 4 of the main text, showing the universality of the epidemic patterns formed by the in-homogeneous SEPIR model.

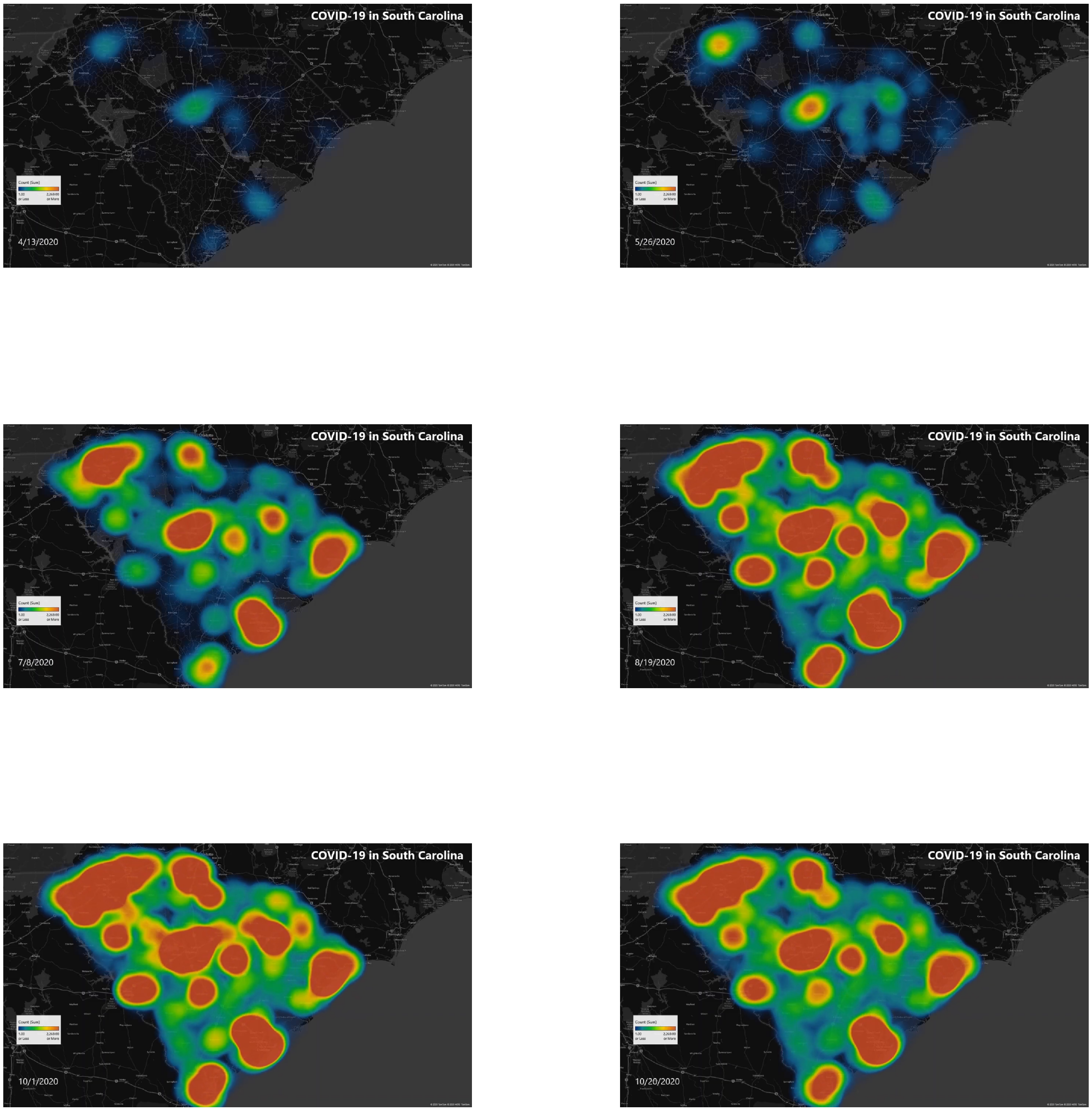

Fig. SI-6. South Carolina heat-maps. The evolution of infection in South Carolina shown as progressing heat-map snapshots of cumulative infections. The snapshots are taken from the movie clip at `https://scdhec.gov/covid19/sc-testing-data-projections-covid-19`. The date is indicated at the low-bottom corner of each image.

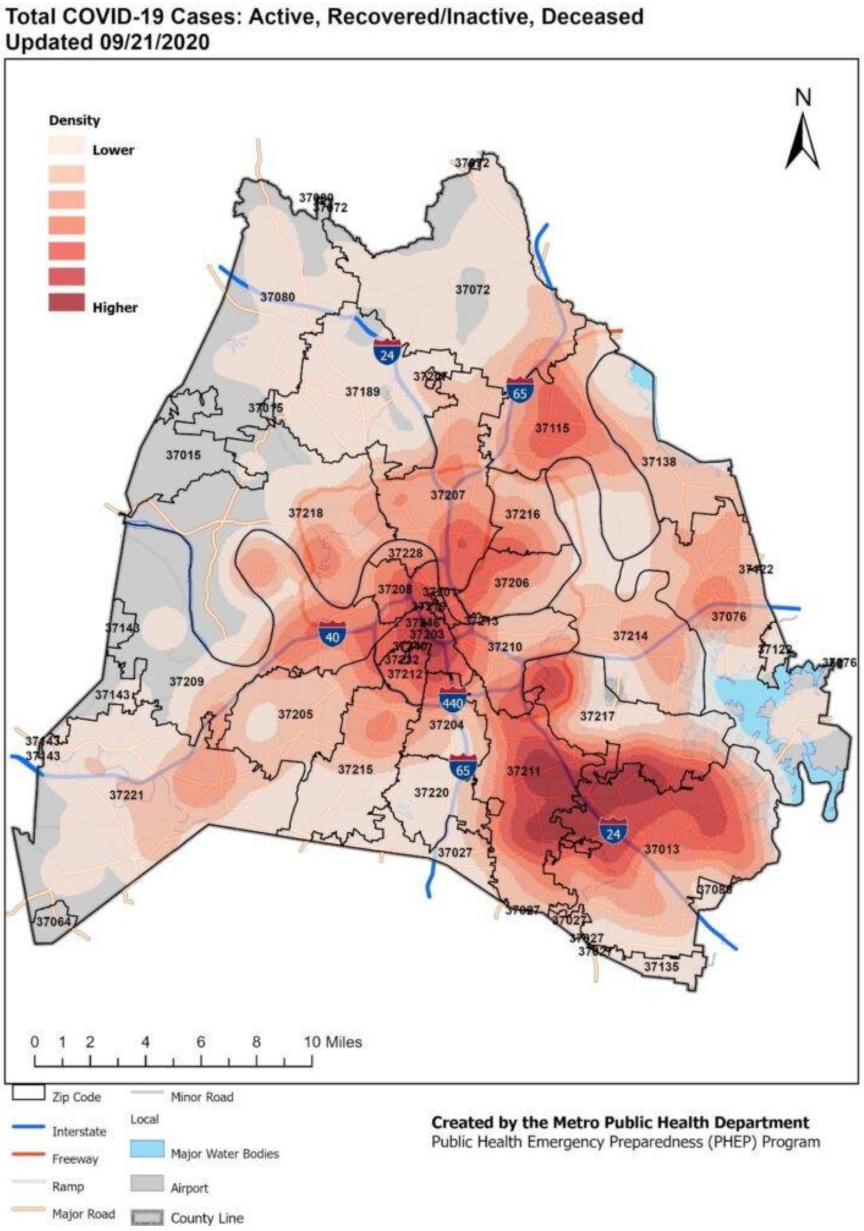

Fig. SI-7. Nashville, Tennessee, heat-maps. Infection heat-map snapshot of the areas of southeast Davidson County and downtown Nashville (Tennessee), from Sep. 21, 2020. For further information see `https://www.wsmv.com/news/davidson_county/heat-map-shows-high-number-of-active-cases-in-green-hills-12-south-areas/article_77bead44-fce4-11ea-856b-e3cd70799d6c.html`
