# Supplementary material for: Epidemiological model for the inhomogeneous spatial spreading of COVID-19 and other diseases
Source: PLoS One. 2021 Feb 19;16(2):e0246056. doi: 10.1371/journal.pone.0246056 (PMC7894958; doi:10.1371/journal.pone.0246056)
Supplement: Attachment — Submitted filename: PONE_Rev_Model for the inhomogeneous spatial spreading of COVID19.docx [file pone.0246056.s002.docx]

The authors investigate a topic of great interest right now, which is how spatial heterogeneity in population density, infection origins, and quarantine measures affects the spread of COID-19 throughout space. The authors extend the basic SEIR framework into a reaction-diffusion model to capture these elements. The extension of the original SEIR framework to incorporate these elements appears robust and the heat maps generated to demonstrate the effect of this heterogeneity on disease propagation through space are attractive and quite interesting.

My most important comments are two-fold:

First, because there is no attempt to ground this model using a practical scenario with real-life data on the population density of a given city or time-dependent quarantine measures of a city, the article is best thought of as a theoretical framework. I find that there is value in the presentation of such a theoretical framework. However, the article falls short of justifying through their results: 1) why this model captures COVID-19 dynamics better than the simple SEIR model with a pre-symptomatic compartment; and 2) how this model either a) improves our understanding of COVID-19 dynamics or b) might reveal which quarantine strategies are most useful.

Second, I would like to see more justification or a sensitivity analysis on a few model assumptions (a) why infection can only occur between nearest neighbors; b) equal transmission between members of the same node and members of neighboring nodes).

In addition, I also have some specific comments below.

**Introduction:**

General:

Since this model is not fit to data, nor does it attempt to capture the quarantine patterns or density of a particular area, I suggest that it be clear that the objective is to present a theoretical framework.

Specific:

- Line 21: The authors observe that the fitted model yields accurate predictions. Is this to suggest that the multi-population extension of the model improves upon the earlier model of Wu, et al?
- Line 22: How do models by He and Giordano improve the original SEIR model in a way that’s relevant to this paper?
- Line 35: The idea of sub-exponential or power-law growth of an epidemic is very compelling. However, as written, the reader must already have some exposure to the idea of “power-law growth” to fully understand this paragraph. I suggest providing a brief definition of “power-law behavior” as well as “quasi power-law” to help with readability. I also suggest a brief explanation of the reason for this power-law growth, drawing on the idea that heterogeneous mixing can lead to this approximation. The cited source 22 has a nice example of this. This can also be a good place to set the stage for how readers should interpret the power-law exponents solved and presented in the results (see my later comment).
- Line 37: Source 22 appears to be missing the journal
- Line 37: Does “such a temporal behavior” refer to power-law behavior?
- Line 38: The wildfire analogy is useful to visualizing “front propagation”, but I suggest providing a brief definition of “front propagation” that can be understood by a wide audience.
- Line 46: “the spreading of disease from areas of low density to areas of high density”
- Lines 47-49: I find that the novelty of this model derives from the inclusion of spatial heterogeneity in spreading, population density and quarantine. The fourth point (inclusion of an asymptomatic compartment) is fairly standard among COVID-19 SEIR models.
- Line 53: suggest to remove “(yet generalized to five populations)” since these five populations haven’t been introduced yet.

**Methods:**

General:

- Both square and hexagonal lattices are mentioned. Which are used for the purposes of this model?
- Nodes are considered to be homes, apartments or areas (line 79), yet it seems from the representation of “cities” that areas may be a more appropriate way to think about the nodes in this particular paper. This may also free the authors from having to justify how k_1_ and k_2_ could be equal if the nodes were homes, and how infection can only spread between nearest neighbors (see my comments under discussion). Would it be correct to represent the nodes here as areas, and allude to the fact that this model could be extended for different contexts for the nodes to be homes?

I also suggest including additional details in the methods section:

- Some of the results text seems like it would be more appropriate for methods. For interest, the first and much of the second paragraph of the result section.
- It would help the reader to have an outline of the different scenarios that will be tested (e.g. 1) uniform density + uniform initial conditions; 2) uniform population density + nonuniform, random, initial conditions; 3) uniform population density + nonuniform, clustered, initial conditions; 4) nonuniform population density with infection originating from inside city; 5) nonuniform population density with infection originating randomly in the city periphery; 6) nonuniform population density with infection originating at two centers in the city periphery; 7) belt quarantine around a city…..ETC.
- The objectives/outcomes of interest for each scenario (e.g. whether it’s herd immunity, time to herd immunity/peak, and exponents of power-laws, F+R at longest simulation time) should also be mentioned.
- For simulations in which the initial conditions were nonuniform but random, please describe how the random initial conditions were generated. Was a single run done, or were the curves depicted in Figures 4, 6, etc. a composite of different random iterations of the model? If a single run done, can the authors justify that patterns would be similar under multiple iterations?
- Line 221 from results: please describe in the methods, or provide a citation for “large density variations”. In other words, how much larger is the city from the suburb? Are rural areas described here or is it just thought of as city or suburb? It would be interesting to see a density heat map presented, either as another panel in the figures or in the supplement.

Specific:

- Line 56: I find it is more clear to consider five disease stages, rather than five populations. I also suggest to define these five stages up front.
- Line 61: x and t are introduced but not defined. I am assuming it to be space and time, but what are the spatial and temporal resolutions or units?
- Line 62: b(x,t) is often thought of as “exposed” according to the SEIR terminology and may be more interpretable if referred to as such here
- Line 70: please define d, could even define it in line 57 as, “…the geographical region under study, *d* [units] by *d* [units] in area, …”
- Line 85: This phrase does not read well: “independent whether the infectious person is at i and susceptible is at j or vice versa”
- Line 89: It might be more intuitive to use a letter other than K to represent K_0_, K_1_ and K_2_ since there are also lower case k’s
- Line 96: Because I tend to think of the h’s, b’s, etc. as stages rather than populations, it took me a minute to realize the *y*’s were supposed to stand in for the different stages. (e.g. y(x,t) represents h(x,t), b(x,t), etc.). Adding an “e.g. …” much in the same way as was done in line 77 may clear this up.
- Line 100: Defining *z* as the number of neighbors around a central node may be more clear than coordination number of the grid.
- Line 124: please provide a citation for the “hypothesis that people are infecting already about 2-3 days before they show symptoms”
- Line 135: what are the different “parameters” used?

**Results:**

General:

For several simulations, it doesn’t appear that herd immunity has been reached. I am curious to know what would herd immunity be under each scenario had the model been reached. Else, I would like a brief mention of why herd immunity levels should not be the target of interest here. Relatedly, much of the simulations report the value of F + R at the longest simulation time. The longest simulation time chosen feels somewhat arbitrary. I am wondering if the authors can either justify why this is the most interesting outcome of interest, rather than the level of herd immunity and the amount of time it takes to reach herd immunity, or provide those values as well. Finally, can some discussion be provided of how readers should interpret the power-law exponents to justify why these are interesting outcomes?

Specific:

- Line 158: For clarity, “just a single additional population.” Could be changed to “just the addition of a pre-symptomatic stage”
- Figure 2 caption: It may be more clear to say “spatially nonuniform initial conditions” rather than “spatially nonuniform infection centers.”
- Line 218: I would refer to “population heat-maps” instead as “infection heat maps” to avoid confusion that this is population density.
- Figure 7 caption: please make this a stand-alone caption.
- In captions, define t, and give units (days)
- Line 259: “and F + R ' 23%” **with quarantine**

**Discussion**

- Herd immunity for the spatially uniform model is not reached until the population is at 90%. This is much higher than the herd immunity estimated from previous study that is nearer to 1-1/R_0_. Can there be some discussion about the discrepancy in these estimates?
- The idea that spatially heterogeneous populations can lower the herd immunity threshold is receiving more attention of late. In the discussion/conclusion, please discuss how this work complements or build upon on other literature, that has been published or are preprints that pertain to this topic. (see just one example here: <https://www.medrxiv.org/content/10.1101/2020.07.23.20160762v1>).
- Please provide more discussion about several model assumptions.
  - The model assumes that all persons will eventually pass from the pre-symptomatic stage through the symptomatic stage. This is not the case with COVID-19, as we know that there are some people who remain asymptomatic.
  - The model assumes that transfer of infection can only occur between nearest nodes, *i* and *j*. This seems to me an unrealistic assumption. For instance, there are several nodes represented even in one “city”, and individuals might travel to a further away node in the same “city” for work. Can the authors either justify this assumption by explaining how results wouldn’t change if this rule were relaxed, discuss it as a limitation but explain why results would stay the same, or demonstrate how infections would propagate if this assumption were relaxed?
  - k_1_ and k_2_ are assumed to be equal (lines 131-133). Do you feel this is a realistic assumption, especially given that the nodes could be thought of as households (line 79)?
  - The quarantine/lockdown measures reported here are quite strict (80% reduction) and in place from t=0 until the end. This is not the case for the quarantine measures for COVID-19.
- Limitations of the model should be discussed.
- Given that this is a framework, yet no specific example pertaining to a geographic area of interest presented, can the authors attempt to justify why this particular model is superior to the spatially homogenous SEIR model? For instance, can the authors demonstrate how the heat maps from the spatially-heterogenous model are more similar to COVID-19 heatmaps as compared to the spatially-homogenous model? Assuming that the authors can demonstrate the superiority of this model over the original SEIR model, what might we learn about COVID-19 dynamics from this model (e.g. infection gravitates moves towards city centers, the benefits of a lockdown vs. ring quarantine, etc.)
