# Supplementary material for: Epidemiological model for the inhomogeneous spatial spreading of COVID-19 and other diseases
Source: PLoS One. 2021 Feb 19;16(2):e0246056. doi: 10.1371/journal.pone.0246056 (PMC7894958; doi:10.1371/journal.pone.0246056)
Supplement: Attachment — Submitted filename: response_to_reviewers.docx [file pone.0246056.s003.docx]

We are most grateful to the three reviewers for their very constructive remarks that helped to improve the manuscript and make it comprehensive to a wide readership.

Below we respond to each reviewer (in red color), and describe the changes made to the manuscript (when appropriate). These changes are also marked in the newly submitted manuscript in blue color.

**Response to Reviewer #1**

The authors investigate a topic of great interest right now, which is how spatial heterogeneity in population density, infection origins, and quarantine measures affects the spread of COID-19 throughout space. The authors extend the basic SEIR framework into a reaction-diffusion model to capture these elements. The extension of the original SEIR framework to incorporate these elements appears robust and the heat maps generated to demonstrate the effect of this heterogeneity on disease propagation through space are attractive and quite interesting.

Response:

We thank the referee for carefully reading our paper and for his suggestions to improve it.

My most important comments are two-fold:

First, because there is no attempt to ground this model using a practical scenario with real-life data on the population density of a given city or time-dependent quarantine measures of a city, the article is best thought of as a theoretical framework. I find that there is value in the presentation of such a theoretical framework. However, the article falls short of justifying through their results: 1) why this model captures COVID-19 dynamics better than the simple SEIR model with a pre-symptomatic compartment;

Response:

First, there is no possibility whatsoever to obtain, from the homogeneous mixing models, heat-maps such as those appearing now on-line in South Carolina and occasionally in other countries. It is clear that in most countries health authorities posses' all the required data to produce such heat-maps, although they decide not to make them publicly available. Such heat-maps are our main predictions.

Moreover, there is a colossal failure of the predictions of epidemiological models (in Israel and worldwide), leading to serious public as well as authority mistrust. We believe that one of the major problems with all these *homogeneous* models, be it SIR, SEIR, or other many populations (compartments) models, is their inability to account for the spatial spreading of the epidemic. The latter yields a much slower evolution. We did explain this in the introduction and throughout the paper. Thus, when one compares Fig. 1 (homogeneous SEPIR model, as we term it now) to all other figures (our inhomogeneous SEPIR model), it is clear that the evolution depicted by our model is much slower.

Furthermore, whereas the evolution of the homogeneous SEPIR model depicted in Fig. 1 suggests "herd immunity" at about H=0.37, or fraction of immune population 1-H=0.63 (indeed very close to the known SIR result, $1-(1/R_{0})$, for the value used in this work $R_{0}=2.5$), the "herd immunity" value that could have been inferred from Fig. 4, i.e. based on the *in*homogeneous SEPIR model, is about 1-H=0.35. This may give a wrong interpretation to observed epidemic curves in various countries where travel restrictions have been imposed. In other words, if authorities conclude from an observed epidemic curve (looking at the peaks of either "daily new infections" or that of "actively infected people") that herd immunity has been reached, and so remove the travel restrictions, they might find that no such herd immunity exists anymore. This is equivalent to putting "by hand" a higher value of $R_{0}$ (in the homogeneous SEPIR model); however, the important difference is that we do not have to guess a new value for $R_{0}$ when travel restrictions are removed; its local value remains the same in all cases studied in the work (except when considering localized quarantine/lockdowns) whether we use the homogeneous SEPIR or the inhomogeneous SEPIR model.

The following two paragraphs (3^rd^ and 4^th^ paragraphs) have been added to the Conclusions section:

"Clearly, the homogeneous (i.e. prefect mixing)… … confirming our model conclusions."

and 2) how this model either a) improves our understanding of COVID-19 dynamics or

Response:

We believe that after the current revision, we made it much clearer, and we hope the referee agrees.

b) might reveal which quarantine strategies are most useful.

Response:

In this paper we considered three quarantines scenarios: (i) Belt quarantine protecting a local area from the surrounding, (ii) Belt quarantine protecting the surrounding from a local region, and (iii) Area lockdown to protect the surrounding from a local region as well as for protection of the local region itself.

To further emphasize these strategies, we now devote a separate subsection ("Quarantine strategies") for quarantine strategies. In addition we emphasize the main difference that we found in the following phrases (at the end of the ("Quarantine strategies" subsection, just before the conclusions):

"Thus, it is clear that area lockdown should be the preferred choice… …If such a careful orchestration is not performed, localized quarantine strategies may have a limited impact."

Second, I would like to see more justification or a sensitivity analysis on a few model assumptions (a) why infection can only occur between nearest neighbors;

Response:

Indeed in normal life conditions people travel long distances, which may create new infection centers, as seen in the South Carolina heat-maps. Once traveling is reduced, the growth will become more local. It should be noted that the term "local" refers to the choice of the grid size, i.e. if people still travel over distances of 30 km, this is the right grid size choice to be used for the "nearest-neighbor" assumption, for which the current model is valid. The proper use of our model should be in regard to restrictions or normal life habits. Infections resulting from long distance travels, or temporal mass gatherings of people coming from distant locations, can be added to our model, but are out of the scope of the present work.

To improve our explanations, we added the following paragraph (3^rd^ paragraph) to the section "Inhomogeneous SEPIR model":

"In order to develop the spatial epidemic spread model,… …traveling may be restricted to 1 km, which

implies $\delta\simeq1$ km."

b) equal transmission between members of the same node and members of neighboring nodes).

Response:

We *do* distinguish in principle between infections rate of the same node ($k_{1}$) and those between nearest-neighbor nodes ($k_{2}$). It is true that in order to estimate the values of the continuous model parameters, we used for simplicity the estimated upper bound of $k_{2}$ ($k_{2}=k_{1}$). Following the referee's remark, we present a sensitivity analysis in the SI that include lower ratios of $k_{2}/k_{1}$.

In addition, I also have some specific comments below.

**Introduction:**

General:

Since this model is not fit to data, nor does it attempt to capture the quarantine patterns or density of a particular area, I suggest that it be clear that the objective is to present a theoretical framework.

Response:

We added the term "theoretical framework" in the (one before the last) paragraph of the Introduction.

Specific:

- Line 21: The authors observe that the fitted model yields accurate predictions. Is this to suggest that the multi-population extension of the model improves upon the earlier model of Wu, et al?

Response:

We believe so. For example, the work from Italy (Giordano et al., Ref. 20) appears to better describe the evolution (and, as far as we know, on the basis of this work an advice was issued to the Italian government how to "reopen" the economy). However, we do not wish judge previously published works. Time will tell what is the best model to use.

- Line 22: How do models by He and Giordano improve the original SEIR model in a way that’s relevant to this paper?

Response:

Our theoretical framework allows to improve any multi-population (multi-compartment) models, such as SIR, SEIR, our homogeneous SEPIR model, and the models used by He et al. and Giordano et al. We refer to these models (in this revised version) as the *homogeneous* models, and the addition of spatial dependence lead to *in-homogeneous* models of the same kind. Since the infection term is equivalent in all these models, the generalization to inhomogeneity is straight forward.

- Line 35: The idea of sub-exponential or power-law growth of an epidemic is very compelling. However, as written, the reader must already have some exposure to the idea of “power-law growth” to fully understand this paragraph. I suggest providing a brief definition of “power-law behavior” as well as “quasi power-law” to help with readability. I also suggest a brief explanation of the reason for this power-law growth, drawing on the idea that heterogeneous mixing can lead to this approximation. The cited source 22 has a nice example of this. This can also be a good place to set the stage for how readers should interpret the power-law exponents solved and presented in the results (see my later comment).

Response:

We added a definition of a power-law in the 5^th^ paragraph of the Introduction, then when we first discuss the results (regarding Fig. 1, 3^rd^ paragraph of subsection "Study of model behavior"), clarifying the mathematical definition of a power-law. The term "quasi linear" was deleted from the abstract, and we left only the terms "sub-exponential" and "power-law".

While we cannot explain by hand-waving all power-law exponents that we obtain numerically, the simple case depicted in Figs. 2, leading an exponent nearly 2, is explained in terms of a front of an infected domain moving at constant velocity. It is possible that we did not explain clearly the reason for this exponent, hence we modified the text to (see end of 4^th^ paragraph of subsection "Study of model behavior" discussing Fig. 2):

" The latter exponent may be explained… …cumulative number of infected people, grow as $\sim t^{2}$."

We modified the discussion in the introduction (3^rd^ paragraph from last) regarding the power-laws and their possible origin:

"Specifically, these models give a broad regime of exponential growth… …which gives further motivation to the present work."

In addition, we added (Conclusions, 3^rd^ paragraph) some discussion of observed power-laws (using analyses of previous works) to relate them to the power-laws obtained in this work:

"Hence, observed deviations from the homogeneous models… …has been deduced for China and Iran [28]."

Line 37: Source 22 appears to be missing the journal

Response: We updated the reference from an arxiv publication to publication in Physical Biology.

- Line 37: Does “such a temporal behavior” refer to power-law behavior?

Response: Yes, this is now clarified.

- Line 38: The wildfire analogy is useful to visualizing “front propagation”, but I suggest providing a brief definition of “front propagation” that can be understood by a wide audience.

Response: We changed the phrasing to: "The first is derivation of a spatial spreading (diffusion-like) operator, that generates propagation of the front of an infected population domain into

a susceptible one [31-33]."

- Line 46: “the spreading of disease from areas of low density to areas of high density”

Response: Corrected according to the suggestion.

- Lines 47-49: I find that the novelty of this model derives from the inclusion of spatial heterogeneity in spreading, population density and quarantine. The fourth point (inclusion of an asymptomatic compartment) is fairly standard among COVID-19 SEIR models.

Response: Correctly so. We changed the text from "four" to "three" major aspects. Nevertheless, it was important to note the extra population for reader that are much familiar with the SEIR model, but not with its multi-population extensions related to COVID-9, see corrected text (one before the last paragraph of the Introduction).

- Line 53: suggest to remove “(yet generalized to five populations)” since these five populations haven’t been introduced yet.

Done.

**Methods:**

General:

- Both square and hexagonal lattices are mentioned. Which are used for the purposes of this model?

Response:

The general derivation of Eq. (3) is done for both lattices, and the value of the coordination number *z* appears in the expressions for $k$ and $D_{k}$ (see the discussion following Eq, (3)). Square lattice is used for the numerical study (using *z*=4), as explained in the text (one paragraph before the last one in section "Inhomogeneous SEPIR model").

Nodes are considered to be homes, apartments or areas (line 79), yet it seems from the representation of “cities” that areas may be a more appropriate way to think about the nodes in this particular paper. This may also free the authors from having to justify how k_1_ and k_2_ could be equal if the nodes were homes, and how infection can only spread between nearest neighbors (see my comments under discussion). Would it be correct to represent the nodes here as areas, and allude to the fact that this model could be extended for different contexts for the nodes to be homes?

- Response:
- Indeed, we completely agree with the referee that "areas" are more appropriate, hence we changed the explanation and defined the "grid size" (of the area units) when we start to develop the model, according to the travel restrictions. We decided to avoid the discussion on "homes" altogether since it will probably confuse the reader. We have already responded to a comment above with the following corrected text (3^rd^ paragraph to the section "Inhomogeneous SEPIR model"):
- "In order to develop the spatial epidemic spread model,… …traveling may be restricted to 1 km, which implies $\delta\simeq1$ km."

I also suggest including additional details in the methods section:

- Some of the results text seems like it would be more appropriate for methods. For interest, the first and much of the second paragraph of the result section.
- Response:

We do not have a "Methods" section; the section that can be associated with "methods" is called now "Inhomogeneous SEPIR model" (name changed). We do not wish to declare a single paragraph as "Methods" section, also we do not wish to append it to the "Inhomogeneous SEPIR model" section. However, in the spirit of this comment we now split the "Results" section into two subsections: "Study of model behavior", and "More complex scenarios". This way, it becomes clear that we first show the characteristic behavior resulting from our model, before analyzing more complex and realistic scenarios. The first paragraph the referee mentions now appears as the introduction to these two subsections.

- It would help the reader to have an outline of the different scenarios that will be tested (e.g. 1) uniform density + uniform initial conditions; 2) uniform population density + nonuniform, random, initial conditions; 3) uniform population density + nonuniform, clustered, initial conditions; 4) nonuniform population density with infection originating from inside city; 5) nonuniform population density with infection originating randomly in the city periphery; 6) nonuniform population density with infection originating at two centers in the city periphery; 7) belt quarantine around a city…..ETC.
- Response:

To follow the referee suggestion we included Table I that summarizes all the different scenarios considered. We refer to Table I in the last sentence of the first paragraph of the "Results" section.

- The objectives/outcomes of interest for each scenario (e.g. whether it’s herd immunity, time to herd immunity/peak, and exponents of power-laws, F+R at longest simulation time) should also be mentioned.

Response:

After the reorganization of, and modifications to, the "Results" section, and the substaintial rewriting the description of the results, we hope that the objectives and outcomes of each of the scenarios is much more clear.

- For simulations in which the initial conditions were nonuniform but random, please describe how the random initial conditions were generated. Was a single run done, or were the curves depicted in Figures 4, 6, etc. a composite of different random iterations of the model? If a single run done, can the authors justify that patterns would be similar under multiple iterations?
- Response:

We accordingly added in the Figure Captions of all relevant figures how the random initial conditions were generated.

Our results are indeed for a single run to mimic how an outbreak can form and spread.

As in other PDE's (e.g., the diffusion equation), if we were to choose a large number of runs with different (randomly chosen) initial conditions, and then average over all results (i.e. average over the results of single runs) we would have obtained a result similar to uniform initial conditions. However, this does not suggest how does a single outbreak look like.

- Line 221 from results: please describe in the methods, or provide a citation for “large density variations”. In other words, how much larger is the city from the suburb? Are rural areas described here or is it just thought of as city or suburb? It would be interesting to see a density heat map presented, either as another panel in the figures or in the supplement.

Response:

The phrase "large density variations" appears in line 211 (of the previous submission). The actual density function (a Gaussian), with all its parameters, was given both within the text and in all the figure captions, from which one can easily tell, for example, that the "city center/core" is 10 times more dense than its "suburbs" or "outskirts". This function also defines the "core" of the city. In order to better explain the implications of this function to non-mathematician, we added a sentence in the text:

"The density in the ``city center", $\boldsymbol{x}\simeq0$, is therefore 10 times higher than in its ``suburbs", and its main core spans over a radius of 10."

Indeed, another "heat-map" for the population density would be worthy for visualization purposes, yet, there are issues concerning the length of the paper, and therefore we added such a heat-map in the Supporting Information (Fig. SI-1)

Specific:

- Line 56: I find it is more clear to consider five disease stages, rather than five populations. I also suggest to define these five stages up front.

Response:

We find that using the suggested definition will be more confusing. Another term that has been suggested to us is ``compartment", which also prefer not to use. Therefore ee decided to change the term "population" to "sub-population".

In the beginning of the section " In-homogeneous SEPIR model" we write:

"Our model, which builds on the homogeneous SEIR model, includes five sub-populations associated with different stages of the disease: susceptible-exposed-presymptomatic-infectious-recovered (SEPIR) ", which we find sufficient.

Since we mention the term sub-population also in the Introduction, we added, in brackets, a similar explanation of the definition:

"(where the term sub-population refers to people under a certain stage of the disease) "

- Line 61: x and t are introduced but not defined. I am assuming it to be space and time, but what are the spatial and temporal resolutions or units?

Response:

We now explain (in brackets) that $\boldsymbol{x}$ is a 2D spatial vector (i.e. coordinate), whereas – not in bold – $x$ is the $x$-component of the vector $\boldsymbol{x}$, and t is the time. As explained at the end of the model section (before Eq. (4)), we also introduce *dimensionless* spatial coordinates, i.e. normalized by the grid-size $\delta$, i.e., $\tilde{\boldsymbol{x}}=\boldsymbol{x}/\delta$. Later, at the end of this section, we clarified that in order *to simplify notations* we rewrite the dimensionless variables without the tilde: $\tilde{\boldsymbol{x}}\to\boldsymbol{x}$. Therefore, to go back to coordinates with dimensions in all our heat-maps it is clear that one should multiply by $\delta$. Regarding the time we use in all figures, it is in units of days, as we now note both on (x-axis labels) and in the figure captions.

We added the following phrase:

"where $\boldsymbol{x}$ is a 2-dimensional (2D) vector in the plane, whose components will be denoted by *x* and *y*"

- Line 62: b(x,t) is often thought of as “exposed” according to the SEIR terminology and may be more interpretable if referred to as such here.

Response: Right, corrected.

- Line 70: please define d, could even define it in line 57 as, “…the geographical region under study, *d* [units] by *d* [units] in area, …”

Response:

In line 70 (previous version), $d^{2}x$ is the common notation of differential unit area (e.g., dxdy, however, coordinate system independent), and we believe it does not need extra definition since it is under the integral sign. 2D is the short of 2-dimensional and indeed, while it is a common short notation, we now defined it when it is first used.

Line 85: This phrase does not read well: “independent whether the infectious person is at i and susceptible is at j or vice versa”

Response:

We changed the phrase to: " Infection can occur at a rate constant $k_{1}(i)$ when infectious and susceptible people from the same node *i* meet each other, and at a rate constant $k_{2}(j,i)=k_{2}(i,j)$ when meetings occur between an infectious person from node *i* and a susceptible person from a nearest-neighbor (NN) node *j* (i.e. the infection rate, for an infectious person at node *i* and a susceptible one at node *j*, is identical to the rate when the two nodes are interchanged). "

- Line 89: It might be more intuitive to use a letter other than K to represent K_0_, K_1_ and K_2_ since there are also lower case k’s

Response:

We accept this request, and we changed the notations of K_0_, K_1_ and K_2_ to $\gamma_{0}$, $\gamma_{1}$ and $\gamma_{2}$.

- Line 96: Because I tend to think of the h’s, b’s, etc. as stages rather than populations, it took me a minute to realize the *y*’s were supposed to stand in for the different stages. (e.g. y(x,t) represents h(x,t), b(x,t), etc.). Adding an “e.g. …” much in the same way as was done in line 77 may clear this up.

Response: Done.

- Line 100: Defining *z* as the number of neighbors around a central node may be more clear than coordination number of the grid.

Response: Done.

- Line 124: please provide a citation for the “hypothesis that people are infecting already about 2-3 days before they show symptoms”

Response: Done.

The new sentence is:" In addition, there is evidence that people are infecting already about 2-3 days before showing symptoms[44], hence we set $\gamma_{1}^{-1}$=3 days. "

- Line 135: what are the different “parameters” used?

Response: Done.

**Results:**

General:

For several simulations, it doesn’t appear that herd immunity has been reached

Response:

This has been done mainly for presentation purposes. We now modified all relevant figures to include the peak in *F* (or *W+F* when the latter curve is distinguishable from *F*)

I am curious to know what would herd immunity be under each scenario had the model been reached.

Response:

The only reason to mention herd immunity is to show that, while a high value of the basic reproductive is used ($R_{0}$=2.5), the apparent herd immunity obtained – from the peak of F (or W+F) – is significantly larger than $H=1/R_{0}$, i.e. the fraction of infected population is much smaller than 1-$1/R_{0}$. The latter is the prediction of SIR (and nearly that of SEIR). While there is a way to circumvent this problem (as done in practice) when SEIR-like models are used, by fitting the epidemic curve with an effective, global, value of $R_{0}$, our approach does not require that, and therefore has a much higher predictive power under the discussed conditions.

Else, I would like a brief mention of why herd immunity levels should not be the target of interest here.

Response:

Indeed herd immunity is not the main focus. However, we accordingly added the following paragraph in the Conclusions section to discuss herd immunity:

" The evolution of the homogeneous SEPIR model suggests "herd Immunity" at about H=0.37 … …confirming our model conclusions."

Relatedly, much of the simulations report the value of F + R at the longest simulation time. The longest simulation time chosen feels somewhat arbitrary. I am wondering if the authors can either justify why this is the most interesting outcome of interest, rather than the level of herd immunity and the amount of time it takes to reach herd immunity, or provide those values as well.

Response:

Our main motivation was to show the correspondence between the epidemic patterns as depicted by the heat-maps and the resulting epidemic curves, and the end time of simulations was chosen according to the possible interest in the obtained heat-map patterns.

As mentioned, following the referee's comments we now extended the range of the epidemic curves to reach a time substantially passed the peak of *F* (or *W+F)*. However, we did not add or replace heat-map panels, except when they are related to an interesting feature in the epidemic curves (e.g., a delayed peak in case of lockdown, when the epidemic leaks out from the quarantined region and spreads quickly).

Finally, can some discussion be provided of how readers should interpret the power-law exponents to justify why these are interesting outcomes?

Response:

Once we defined in the Introduction, and later in the text, the notion of a power-law growth and emphasized that it is much more slower than an exponential growth, we believe that there is no need to repeat this in the analysis of results. However, obviously the value of the power-exponent is meaningful, i.e. a larger one implies a faster growth. Hence, we added this point in the comparison of power-law exponents (the first time it appears), e.g.:

"We observe a separation of the growth into two (albeit very short) power-law regimes, $1-H\simeq A t^{\nu}$ (where A and $\nu$ are numerical constants) with the early evolution exponent $\nu\simeq0.26$ being much smaller than the late evolution exponent $\nu\simeq4$; a smaller power-law exponent $\nu$ signifies a slower growth."

Specific:

- Line 158: For clarity, “just a single additional population.” Could be changed to “just the addition of a pre-symptomatic stage”

Done. However, as mention we still prefer to use the term "sub-population".

- Figure 2 caption: It may be more clear to say “spatially nonuniform initial conditions” rather than “spatially nonuniform infection centers.”

We changed to: "randomly scattered infection centers". (All figures except Fig. 1 correspond to non-uniform initial conditions.)

- Line 218: I would refer to “population heat-maps” instead as “infection heat maps” to avoid confusion that this is population density.

Right. We changed to "infection heat-maps".

- Figure 7 caption: please make this a stand-alone caption.

Response: The captions of old Fig. 7 (new Fig. 6) should be exactly identical to those of old Fig. 6 (new Fig. 5), except for the difference mentioned. Note that the captions of old Fig. 6 (new Fig. 5) are very lengthy and repeating them will be space consuming, and moreover, we believe it is clearer when we just emphasize the differences. Hence, we prefer not to implement this recommendation.

- In captions, define t, and give units (days).

We added into the label of x-axis (time axis) the time units "[days]", and also noted in the captions that *t* is the time in days.

- Line 259: “and F + R ' 23%” **with quarantine**

Done.

**Discussion**

- Herd immunity for the spatially uniform model is not reached until the population is at 90%. This is much higher than the herd immunity estimated from previous study that is nearer to 1-1/R_0_. Can there be some discussion about the discrepancy in these estimates?

Response:

The referee is perfectly right, this was a wrong statement. "Herd immunity" should be deduced from the peak of the infected population. Indeed our *homogeneous* SEPIR model yields herd immunity (from the position of the peak of $W+F$) at about $1-H\simeq0.37$, close to the SIR model prediction $1-H=0.4$ for $R_{0}=2.5$. As mentioned, we have a new paragraph discussing herd immunity, in which we also discuss its possible miss-interpretation when the evolution is *in-homogeneous*.

- The idea that spatially heterogeneous populations can lower the herd immunity threshold is receiving more attention of late. In the discussion/conclusion, please discuss how this work complements or build upon on other literature, that has been published or are preprints that pertain to this topic. (see just one example here: <https://www.medrxiv.org/content/10.1101/2020.07.23.20160762v1>).

Response: This is an important reference that coincides completely with our model conclusions, and we relate to it in the new paragraph (in the Conclusions section) mentioned above that discusses herd immunity.

- Please provide more discussion about several model assumptions.
  - The model assumes that all persons will eventually pass from the pre-symptomatic stage through the symptomatic stage. This is not the case with COVID-19, as we know that there are some people who remain asymptomatic.

Response:

Indeed, there are about 16%-40% (worldwide) of infected people that remain asymptomatic. It is possible in principle to add them as a separate sub-population.

Yet, their infectious properties are known to be different from the symptomatic people, and not fully determined. For example their infection period is suggested to be longer by 5 days on the average, but their viral load is about half or less of that of the symptomatic, leading to a reduced mean $R_{0}$. Nevertheless, a minority of the asymptomatic have been recently found to have a normal viral load, and, when no social distancing and mandatory masks are imposed, they become super-spreaders (enhanced also by the longer infection period). While knowledge is accumulating, it is still hard to model their role in the infection.

The following phrases have been included:

"… In particular, the asymptomatic population forms about 16%-40% of the total infectious population [52], and its infectious properties are quite different from those of the symptomatic population [53]. As knowledge is accumulating, it would be worthwhile to include into the model the asymptomatics, either as a single population, or as two separate populations, ``asymptomatic-normal" and ``asymptomatic-super-spreaders". "

- The model assumes that transfer of infection can only occur between nearest nodes, *i* and *j*. This seems to me an unrealistic assumption. For instance, there are several nodes represented even in one “city”, and individuals might travel to a further away node in the same “city” for work. Can the authors either justify this assumption by explaining how results wouldn’t change if this rule were relaxed, discuss it as a limitation but explain why results would stay the same, or demonstrate how infections would propagate if this assumption were relaxed?
- Response:

First, the following phrases now appear in the beginning (3^rd^ paragraph) of the " In-homogeneous SEPIR model" section:

"In order to develop the spatial epidemic spread model, we consider first… …traveling may be restricted to 1 km, which implies $\delta\simeq1$ km."

Second, in the Conclusions section we added (in the first paragraph) the following phrases:

"Nodes are defined by the assumption that within each node the frequency of contacts is still uniform, thus following a homogeneous model description. This allows to estimate the lateral (linear) size of a node $\delta$ (i.e. area equal to $\delta^{2}$), and makes our model flexible enough to describe infectious disease spreading on a scale ranging from a neighborhood to a whole country."

- k_1_ and k_2_ are assumed to be equal (lines 131-133). Do you feel this is a realistic assumption, especially given that the nodes could be thought of as households (line 79)?

Response:

We have removed this association of a node as household for this reason.

We did note, in our estimated values, that $k_{2}$ can be less than $k_{1}$ (i.e. $k_{1}$ is its maximum value), and in the sensitivity analysis appearing now in the Supporting Information we allowed it to be smaller than $k_{1}$.

- The quarantine/lockdown measures reported here are quite strict (80% reduction) and in place from t=0 until the end. This is not the case for the quarantine measures for COVID-19.

Response:

We understand this issue. This is assumed here as a case study, as we cannot (in this first publication) cover all scenarios. Obviously, when the (localized) lockdown is lifted too early, the epidemic will spread quickly from the infected area and technically our model allows to predict the outcome of any such event (by ending the simulation at the time of lockdown lifting, and then running the model without the lockdown with the last population distributions used as initial conditions). Currently some countries (e.g., Israel and UK) have a plan for localized quarantine measures, that will be lifted when the quarantined region will become "green".

We have already discussed this in the last pargraph, in which we added the following phrase:

"… For instance, in Israel, a plan has been proposed (``the traffic lights plan'' [56]) to impose differential lockdown measures on ``red cities'', and similar plans have been issued for the UK [57]. Our model allows to simulate the impact of these measures and make comparison with the evolution without intervention. …"

- Limitations of the model should be discussed.

See next response.

- Given that this is a framework, yet no specific example pertaining to a geographic area of interest presented, can the authors attempt to justify why this particular model is superior to the spatially homogenous SEIR model? For instance, can the authors demonstrate how the heat maps from the spatially-heterogenous model are more similar to COVID-19 heatmaps as compared to the spatially-homogenous model? Assuming that the authors can demonstrate the superiority of this model over the original SEIR model, what might we learn about COVID-19 dynamics from this model (e.g. infection gravitates moves towards city centers, the benefits of a lockdown vs. ring quarantine, etc.)

Response:

The long paragraph before the last one, in the Conclusions section, is now extended and relates to all these issues:

"Accurate predictions of COVID-19 heat-maps … using long distance infections, which we defer to future work."

**Response to Reviewer #2**

The authors have presented an interesting mathematical model for COVID-19 that accounts for spatial heterogeneity in population density and population mixing. I believe this type of model can be very useful and an improves upon the SEIR model by representing the spatial dynamics of transmission. However, the manuscript is written in a very technical manner and is not accessible to the general readership of PLOS ONE. In order for this work to be published in this journal, I suggest it be rewritten to be more explicitly relevant to the current COVID-19 pandemic. I have provided specific comments below, but I broadly recommend that the authors better describe how their model and findings improve our understanding of COVID-19 transmission dynamics and relevant interventions. How does the model improve upon other existing models that account for spatial heterogeneity in population density and mixing? How do the specific simulations presented here relate to the real world spread of COVID-19? How plausible is it that the data needed for this model will be available? What are other possible uses of this model in the context of the COVID-19 pandemic?


Introduction

- Lines 32-38: These sentences are unclear and have a lot of terminology that has not been defined. Could you rewrite them defining terms such as power-law growth and “front propagation”. As it stands, I’m unsure what the hypothesis is and how it relates to the spatial mixing discussed above.

Response:

We have changed the text to:

"Specifically, these models give a broad regime of exponential growth of the cumulative number of infected people, whereas the actual growth is sub-exponential, which can be effectively described by a power-law (i.e. $A t^{\nu}$ where A and $\nu$ are numerical constants) [24-28]. Early data from several countries indeed demonstrated such a wide temporal regime of power-law growth, which occurred much prior to the peak of the epidemic [24,28-30]. In accord with similar ideas [24], we hypothesize that such a temporal behavior results partially from spatial growth of infected geographical domains, similar to the spreading of wildfire, which gives further motivation to the present work."

- Lines 49 – 53: These sentences don’t say much about the results and seem to be saying that the model is indeed different from SEIR, which is true by construction. If the authors want to include a sentence describing their results, it should be more specific and describe what is learned from their model in the larger context of transmission dynamics and interventions.

Response:

We have changed the text to:

"Our results provide infection ``heat-maps", reminiscent of those appearing in publicly available resources [35]. These heat-maps demonstrate unique features of the disease spreading depending on the spatial variation of population density, location of the initial epicenters, and local quarantine levels. They show that the accumulated number of infected people deviates significantly from the associated homogeneous model."

- It would be useful to add a section to the introduction about other models that have attempted to account for spatial heterogeneity, quarantine, and heterogeneous mixing. This will allow the authors to make more explicit how their model is novel and/or adds to the existing literature.

Response:

We have added (to the second paragraph of the Introduction) phrases describing previous spatial models, explaining the new part in our model and how our model solves some critical issues in these previous models:

"… Some extensions of SEIR-like models that account for spatial variability employed diffusion processes for the different sub-populations [9-12] (where the term sub-population refers to people under a certain stage of the disease). Yet, clearly, while such processes can effectively describe wildlife motion in some systems, they fail to describe human behavior [13]. One of the most important artifact for the application of diffusion processes for human population is the unrealistic tendency to spread all populations to uniformity. Moreover, these models do not include spatially-dependent infection spreading parameters, which are required to model geographically local quarantine."

Methods:

- Line 115: This section should be updated to include more recent literature estimating these parameters across various populations and time periods. There have been a myriad of studies that estimate these parameters and the authors should provide justification for their parameter value choices.

Response:

We have rewritten the paragraph concerning the parameter estimate, providing more references that support these values. However, it should be noted that these parameters, especially $R_{0}$ which is sensitive to human social behavior, vary significantly between different states or continents, and it is impossible to present the preliminary results of our novel model for all situations. Moreover, the main purpose of the manuscript is to present our model and show its importance for application in the COVID-19 pandemic.

We do provide now in the Supporting Information a sensitivity analysis for a range of parameters.

- Line 124-125: These parameter values are very uncertain, so the authors should use sensitivity analyses or multiple simulations to explore how sensitive the results are to the chosen values.

Response:

Indeed so. We now present in the Supporting Information a brief sensitivity analyses. While quantitatively a variation in the results is highly visible, the qualitative similarities in the spatial evolution patterns are also clear.

- Line 138: I wouldn’t think that the “heat map” data the authors are describing is readily available in many contexts. They should discuss the availability of such data here and in the conclusion, and provide citations and examples of these data, if available.

Response:

Heat-maps are usually available in some states on-line depending on the policies of the local authorities. Indeed, the link to Orlando's COVID-19 heat-maps has been removed from Florida's dept. of Health website (Ref.  29 in the old version), hence it has been removed from the reference list. We have replaced it by different link of South Carolina Health authorities (Ref. 35), in which a number of movies describing heat-map evolution in time appear (these heat-map movies also appears in youtube). Since there is still data lacking to provide comparison between our model predictions and the observed heat-map movies, these movies are provided only for motivation, qualitative comparison, and impression. It is compelling to understand that in this evolving pandemic many available sources do not appear in conventional peer-reviewed journals, and we hope the referee can be sympathetic to this limitation.

- The authors should make it clearer in the introduction that they are demonstrating the use of a new model structure but are not relating it to any real population. That said, why are the authors not focusing on a specific region or population affected by COVID-19?

Response:

We have made it even clearer that due to the lack of geographic data from a specific state, we cannot provide such specific predictions. We are currently trying to obtain the required data from different authorities (Florida, New-Jersey, South Carolina, and Israel), but in light of privacy regulations this is now on hold, and is out of the scope of the present work. Such data should include detailed heat-maps at different periods of the evolution, such as those we refer to by web links in the appropriate references, as well as high resolution geographic density of population.

Hence, the main purpose of our paper is *to present our novel model, that will allow researchers working in close contact with authorities to apply it in their own countries* (as all the required data will be available to them).

Results

- Generally, the results should be written more for a general audience. For each simulation, the authors should make it clear why the examples were chosen and how they relate to real-world transmission situations. What do the results of each simulation tell us about COVID-19 transmission? For example, what would cause each of these simulation scenarios to happen in the real world? Have there been examples of such transmission scenarios the authors can point to? What are the implications of the initial conditions of the simulations for how we interpret the results?

Response:

Following the referee remark, we made extensive changed to the text. We divided the "Results" section into two subsections. The first subsection ("Study of model behavior") is aimed to study the new model properties, by analyzing oversimplified situations. These can help to understand the more realistic situations studied in the second subsection ("More complex scenarios"). While still, as noted, we did not consider initial conditions taken from previous outbreaks and density heterogeneity of a particular region on the globe, we commented on the motivation for studying these example, by referring to heat-maps from initial stages of the epidemic evolution in South Carolina. These heat-maps show a scatter of a few or several hotspots from which the epidemic grows.

- All heatmap figures are hard to understand due to a lack of legend and labeling. The axes (currently X and Y) of each panel need to be labeled and the color scheme needs to be defined and labeled.

Response:

We made many improvements to figure legends. We now clarify in the text: "… where $\boldsymbol{x}$is a 2-dimensional (2D) vector in the plane, whose components will be denoted by x and y", and corrected the axes labels.

- If the authors want to do simulations in a city, why not use real population density data from a relevant city experiencing a COVID-19 outbreak?

Response:

We thank the referee for encouraging us to further search for publicly available data and collaborations under contract with health authorities, despite the high bureaucratic barriers. Overcoming these barriers will consume a lot of time and prevent many modelling groups to apply our model immediately. We have found publicly available heat-maps from South Carolina that are now cited in Ref. [35], and we shall seek for the data used to obtain these maps, as well as the population density variation in this country. This is out of the scope of the present study, and we leave it for future publication. We emphasize that our model can be adopted to different scales, from a single city to the scale of a complete state.

- I think the city analyses are useful and more relevant to real life, but the decisions about how to initiate infections should be explained more in terms of real-life situations. How would these initial conditions occur in the real world?

Response:

Initial infection centers obviously result from in-flux of infections via numerous normal real-life processes, e.g., when infecting people visit the city for a short period, or when infecting and susceptible people meet in different locations outside the city (e.g., an event) and then return home, and so on. Our model commences from these hot-spots, currently without modeling their creation specifically (which can be done by constructing a hybrid mode). Under certain quarantine measures (e.g., blocking transportations between large cities), such processes will be obviously minimized, but the hotspots created within cities will evolve to an infected population domain as we predict and observed in the South Carolina heat-maps referenced above. To clarify this issue, we added the following sentence to the text (sub-section "More complex scenarios", 1^st^ paragraph):

"In real-life, such initial infection centers obviously result from in-flux of infections *via* numerous processes, e.g., when infecting people visit the city for a short period, or when infecting and susceptible people meet in different locations outside the city (creating infection events) and then return home, and so on."

- Are “belt quarantine” and “area lockdown” real strategies used to fight the spread of COVID-19? Please provide examples and citations for these strategies. If they are not common strategies, can the authors include simulations of more commonly used quarantine strategies?

Response:

We added the following discussion to the text which includes examples of "area lockdown" and "belt quarantine", giving the motivation in the spirit of the reviewer's remark and appropriate references (sub-section "Quarantine strategies", 1^st^ paragraph):
"We define local "area lockdown" as strong restrictions on activity within a certain area. Examples where "area lockdown" have been employed are… . We also propose another strategy that inflicts less burden on the society, which we term ``belt quarantine". Here movement restrictions are imposed between a certain region and its surroundings, but not inside the region itself. Partial belt quarantine has been employed for instance in… "

Conclusion

- This section needs more discussion of existing literature and models that account for spatial heterogeneity and estimate the impacts of quarantine on transmission. How do the results from this model/analysis compare with existing literature? How does this paper add to our understanding of quarantine interventions and transmission dynamics?

To the best of our knowledge, spatial models have almost not been used for COVID-19.  There have been a few exceptions. A few papers divided a country to a large number of zones and introduced infection dynamics between them using a large number of infection parameters. One paper (suggested by reviewer #3), now added to the manuscript as Ref. [10],  used a diffusion process of the people themselves, as introduced to describe infection in wildlife, to simulate the infection dynamics in France, and used available heat-maps at a given time to predict the future heat-maps (without comparison with observation). As explained above, there is an inherent error when on adds a diffusion of species themselves (here humans), since this process introduces the spreading of the given population in the whole region to obtain homogenous distribution. Conversely, our deduced diffusion-like operator concerns the spreading of the infection, not the species. We emphasize that now more clearly in the text, e.g., in the Introduction (6^th^ paragraph): "… Importantly, this operator is associated only with infection spreading and does not describe motion of the different populations as previously suggested [ ]; as argued above, the use of a diffusion process is inadequate for human population. …". The very same reference [10] does analyze the effect of lockdown. Our approach is somewhat analogous to this work, using our more realistic approach.

While we do not add a lot to the qualitative understanding of the effect of quarantine, our unique approach allows to quantify it in a more reliable manner than currently possible. This is extremely important for optimization of lockdown measures, especially localized lockdowns in so-called "red cities". On the one hand one needs to use enough measures to defeat the disease; on the other hand, one needs to minimize the negative impact on the society due to these measures, and the quantitative estimate can help to find this delicate balance.

- As mentioned above, the authors need to add more in-depth discussion of the data needed for this model to be operationalized. I’m not familiar with what they call “heat maps” and am not sure how available they will be. Please add some information about what exactly these data are and where they are available.

We have inserted the following paragraph (6^th^ paragraph) in the Conclusions section:

"Accurate predictions of COVID-19 heat-maps will require complete data sets for: ... …; this can be readily done using long distance infections, which we defer to future work."
- It would also be useful for the authors to more explicitly discuss the potential uses of their model in the context of the COVID-19 pandemic.

We added the following sentences to the last paragraph of the Conclusions section:

" For instance, in Israel, a plan has been proposed (``the traffic lights plan'' [56]) to impose differential lockdown measures on ``red cities'', and similar plans have been issued for the UK [57]. Our model allows to simulate the impact of these measures and make comparison with the evolution without intervention."

Minor comment: The authors should edit the writing to make sure the grammar is correct throughout the paper.

Response: We made our best to correct the grammar.

**Response to Reviewer #3**

Page 2 Lines 8-10: "Infectious disease spreading models are largely based on the assumption of perfect and continuous stirring", similar to the one used to describe the kinetics of spatially-uniform chemical reactions. In particular, the well-known susceptible-exposed-infectious-recovered (SEIR) model, builds on this assumption."

This is true in some cases, but is very limited. The term SEIR just refers to the compartments which are used, and many extensions are available in both the deterministic and stochastic literature (the authors note a few). It would be clearer to call this the homogenous-mixing or baseline SEIR model.

Response:

We accept the referee's remark. Indeed the assumption of homogenous mixing is not related to the number of populations (compartments, using the referee's terminology), which we now term "sub-populations" to avoid confusion. We thus made a correction and elaborated accordingly (Introduction, 1^st^ paragraph):

"…In particular, the well-known susceptible-exposed-infectious-recovered (SEIR) model, builds on this homogeneous-mixing assumption." We term our full model now as "inhomogeneous SEPIR model"; the homogeneous version of it is call "homogeneous SEPIR model". (Likewise, one can call the familiar SEIR model "homogeneous SEIR model", which can be similarly generalized to "inhomogeneous SEIR model".)

It seems that more information about how the proposed techniques differ from other deterministic approaches which appear superficially similar. A few examples:

<https://aip.scitation.org/doi/full/10.1063/1.5116807>
<https://link.springer.com/article/10.1007/s11587-013-0151-y>
<https://arxiv.org/abs/2005.03499>

Response:

We have added the following description of spatial inhomogeneity accounted by other models and included the above references (Introduction, 1^st^ paragraph):

"…Some extensions of SEIR-like models that account for spatial variability employed diffusion processes for the different sub-populations [9-12] (where the term sub-population refers to people under a certain stage of the disease). Yet, clearly, while such processes can effectively describe wildlife motion in some systems, they fail to describe human behavior [13]. One of the most important artifact for the application of diffusion processes for human population is the unrealistic tendency to spread all populations to uniformity. Moreover, these models do not include spatially-dependent infection spreading parameters, which are required to model geographically local quarantine."

> Page 2 line 31 "often require refitting the infection rate constant as the epidemic progresses"

I don't believe this should neccessarily be listed as a negative - in real epidemics/pandemics, there is usually very little reason to believe that epidemic intensity (which involves infectiousness as well as contact rates) is constant over space or time. The authors later note this and express the idea more clearly, but I think this could be clarified.

Response:

The referee is absolutely right. Human behavior changes during an epidemic, even without any social distancing measures, which implies that $R_{0}$ is changing (even if slowly) during the epidemic evolution. The referee is also right that $R_{0}$ may vary from one area to the other, for example, if people behave differently across these areas. Indeed, any spatial dependence of $R_{0}$ is accounted for in the spatial dependence of the two model parameters *k(****x****)* and *D(****x****)* (they are both proportional to $R_{0}$).

Accordingly, we changed the phrase to (Introduction, 4^th^ paragraph):

"… Moreover, the basic reproductive number, $R_{0}$, may vary from one area to the other, for example, if people behave differently across areas. A further complication may arise when human behavior evolves in time during an epidemic, implying that $R_{0}$ is also time-dependent. As a consequence, accurate predictions of such models rely on repeated readjustments of the infection rate constant as the epidemic progresses. "

I think the manuscript would be greatly enhanced by a more detailed explanation of the degree of model fit, and any appropriate sensitivity to the driving parameters.

Response: We added sensitivity analysis to the model parameters, see Supporting Information.

In addition, the discussion should address the following issues:

1. Application of this technique to areas of sparse spread/inflated zero counts

Response: We are not sure what the referee means by this remark. Most of our examples use as initial conditions few/several localized infected population *b(****x****)*, such that indeed "zero counts" appear in most of the area under study (contrary to homogeneous models where they are absent).

2. Limitations concerning the use of incomplete testing data for validation

Response:

This is an important issue for all models or any analysis of disease evolution. It is well known (from previous infections as well as from the COVID-19 pandemic) that testing data accounts between 2.5%-20% of the positives (depending on the testing policy in each state/country); Based on various serological polls, COVID-19 positives in western countries usually accounts for about 10% (or 5%-15%) of the true infected population. This however is not going to severely hamper our predictions so long as the incomplete testing data **is uniform in space** (e.g., 10% everywhere), and we believe that in general this is so within a certain country. Obviously, absolute predicted numbers will not be obtained without the use of the required adjusting factor between tested and predicted numbers.

We therefore added the following sentence in the CONCLUSIONS section, one paragraph before the last one:

" Incomplete testing data is not going to severely hamper our predictions so long as it is uniform in space (e.g., only 10% of the COVID-19 positives are detected everywhere), and in general we may expect this to be so within a certain country where a uniform testing policy is adopted. Obviously, absolute predicted numbers will not be obtained without the use of an adjusting factor between tested and predicted numbers. "

3. People travel great distances, which would seem to limit the utility of this model. Can it be applied to large areas where non-contiguous spreading events occur, or would that require generalization?

Response:

The referee is right that under completely normal modern life conditions, people travel great distances, thus promoting spread of the epidemic to distant locations and creating new hotspots. We did not include such far-distance infection kinetics, and this will be dealt with in future work. We note however that when epidemics evolve, traveling may be reduced due to restrictions or just change of habits.

We therefore added the following sentence in the CONCLUSIONS section, end of the paragraph before the last one:

"It should be noted that when normal life conditions are restored, our model has to be modified to include far-distance traveling; this can be readily done using long distance infections, which we defer to future work."
