# Supplementary material for: Epidemiological model for the inhomogeneous spatial spreading of COVID-19 and other diseases
Source: PLoS One. 2021 Feb 19;16(2):e0246056. doi: 10.1371/journal.pone.0246056 (PMC7894958; doi:10.1371/journal.pone.0246056)
Supplement: Attachment — Submitted filename: response_to_reviewers_2.docx [file pone.0246056.s004.docx]

We are grateful to the reviewers for their constructive remarks that helped to improve the manuscript. Below we respond to each reviewer (in red color), and describe the changes made to the manuscript. These changes are also marked in the newly submitted manuscript in blue color.

Reviewer #1: The authors have put forth a great effort to address the comments of the reviewers, and should be commended. In particular, I appreciate the addition of the supplement as well as the clear outlining of quarantine strategies and model scenarios. I have some remaining minor comments, regarding clarity:

Introduction:

Substantial edits were made to the introduction in order to make the work more accessible to the general readership. However, some improvements could still be made to improve readability. In particular:

- Line 12 "Some extensions of SEIR-like models that account for spatial variability employed diffusion processes for the different sub-populations". It is unclear to me here what specifically is diffusing. I also don't think the reference to wildlife is particularly helpful. One suggestion-- these sentences might be replaced with sentences such as: "Some extensions of SEIR-like models account for spatial variability by employing diffusion processes on contact networks, in which an infection that appears at one node of a network spreads throughout the system through inter-node transmissions. However, this diffusive movement of disease through a population fails to capture human disease dynamics as human movement is not uniform".

Response:

We have added the following sentence:

"To mimic human behavior more realistically, recent extensions employed diffusion processes of the sub-populations that are limited to contact networks [14]"

- Line 21: is it possible to replace “such” with “homogenous”? I think it would be helpful to have this term introduced early on since it is highlighted throughout.

Done

Results/Abstract:

- I appreciate the additional comparison of the infection heat maps to data from South Carolina. However, while reference to South Carolina appears in the abstract, no mention of it is in the text (with the exception of its reference in lines 70, 435, 475, 478). When referenced, it would be helpful if it would be clear that the "publicly available resources" or "heat maps appearing online" is from South Carolina.

Done.

- Relatedly, the citation for [35] appears to be annotated. I also was unable to visit the link and received an error. I understand there are challenges with changing links and the authors should not be held to this. If, however, it is possible to screenshot some instances of the video heat map to include in the supplement, this could alleviate this issue.

We added to Fig. SI-6 (in the SI) six snapshots of COVID-19 heatmaps from South Carolina, with the web-link to the video in the figure caption.

We corrected the technical issue with the link, so now it works.

Discussion:

- The authors now use "sub-populations" to refer to the compartments of the SEPIR model. However, in many places (e.g., paragraph starting at line 422; paragraph starting at line 468) “populations” are still used rather than “sub-populations”

Corrected.

- Line 457: “supporting” our model conclusions, rather than “confirming” our model conclusions

Corrected

- Line 492: define “red cities” instead of using jargon (e.g. "cities labeled 'red' for the highest risk tier")

Corrected to "cities with strong outbreak"

Reviewer #2: The authors have carefully and successfully responded to my initial comments, and I believe the manuscript is much stronger. I only have a few minor remaining comments:

Line 51: Can you reword the hypothesis? Specifically, what are you referring to with “spatial growth of infected geographical domains”? Can you use more general language to make this clearer?

The phrase is changed to: "… we propose that such a temporal behavior can result from the lateral spreading of infected domains, similar to the spreading of wildfire, which gives further motivation to the present work."

Line 69: What sorts of publicly available resources are you referring to here? It would be good to be more specific about the data products you are emulating and how those data can be used. A few specific examples such as the South Carolina Health department data would be useful in the text.

We added: "(e.g., for South Carolina [37] and Nashville, Tennessee [38]; see also the heat-map snapshots in the SI, Figs. SI-6 and SI-7)"

We also added Fig. SI-7 to the SI where we show a heat-map snapshot from Tennessee with an appropriate link (as in Ref. 38) in the figure caption.

Line 11: I like this added text to provide context for the model but I think it could still use more detail. For example, as it is written, it isn’t clear why diffusion processes “fail to describe human behavior”. Could you describe why this is inappropriate more fully?

Text changed to:

"…they fail to describe the (non-random) human behavior [13]. To mimic human behavior more realistically, recent extensions employed diffusion processes of the sub-populations that are limited to contact networks [14].

I appreciate the added sensitivity analyses for parameter values. Could you add a description of the results to the main text? What do you mean by “qualitative similarities” in the results? It would be good to describe specifically how the results change with different specified parameter values.

We feel that providing too many results in the main text will disrupt the continuous reading and will make the paper incomprehensible for of a wide readership. Hence we would like to keep the full sensitivity analyses to the SI.

However, we added the following description to the main text (and added Fig. SI-5 to the SI):

" Sensitivity analysis for a wide parameter range is performed in the Supplementary

Information (SI); see Figs. SI-3, SI-4, and SI-5. As can be expected, the speed of epidemic spread is highly sensitive to the model parameters. Yet, the spatial epidemic spreading patterns that are obtained are quite similar to one another in the range of chosen parameters, e.g., a larger *R_0* pattern appears similar -- though at shorter times -- to a smaller *R_0* pattern. "

We also added to the paragraph describing Fig. 4:

"In Fig. SI-5 we use identical initial conditions as in Fig. 4 but with a value of *R_0* that is twice larger -- *R_0*=5. The snapshots in Fig. SI-5 are taken at shorter times, which yields patterns almost indistinguishable from those shown in Fig. 4, and shows the universality of the epidemic spreading patterns formed by the SEPIR model."

I like the statement that “the main purpose of the paper is to present our novel model to allow researchers working in close contact with authorities to apply it in their own countries” and believe it should be placed at the end of the Introduction section to make it clear what the scope of the paper is.

We have composed a new sentence (1st sentence, last paragraph of the Introduction) :

"In this paper, we develop a novel theoretical framework to model the spread of infections, thereby allowing researchers working in close contact with authorities to apply the model in their own countries."

Table 1 is very useful to guide readers. Could the authors relabel the second and third columns using words? E.g., “Population density, n” and “initial infections” or something similar.

Done.

The figures and legends are much clearer now, but I am still unsure why the displayed time steps are different for each figure? I understand there are space constraints but maybe the authors could justify the choice of displayed time steps. I would also make sure that “x” and “y” are clearly defined in all figure legends.

The times steps are chosen in conjunction with the epidemic spreading patterns that emerge. The different conditions in each study determine the speed of the epidemic spreading, and we see no point to show a number of heat maps with little difference between them.

We have repeated the coordinate definitions "x" and "y" in all figures as suggested.

There remain some grammar and spelling mistakes in the text. It would be good to carefully review.

Done.

Reviewer #3: The authors have made a number of important improvements, and clarified some of the issues which were brought up. There are some remaining issues to be addressed, however.

There are still some problematic claims about the existence of spatially heterogeneous techniques (e.g., "Moreover, these models do not include spatially-dependent infection spreading parameters, which are required to model geographically local quarantine.")

1. Metapopulation models routinely allow spatially-dependent infection parameters.

<https://www.sciencedirect.com/science/article/pii/S0022519396900429>

Part of the objection here is to the strength of the language used describing the limitations of existing models (particularly since the authors don't really engage the stochastic modelling literature much). Using a rich spatially heterogeneous parameterization of a compartmental epidemic model is not terribly difficult to do from a forward-simulation perspective, but often becomes computationally impractical when attempts at fitting a model to actual data are made (especially where formal inference is desired). It is certainly true that the vast majority of work published recently on COVID makes problematic assumptions like homogeneous mixing, but that's largely not a theoretical problem, but a practical one. This distinction should be clearer.

We have inserted the word "naturally" in the sentence mentioned above, and added another sentence:

”Moreover, these models do not involve **naturally** a spatial dependence of infection spreading parameters, which are required to model geographically local quarantine. Thus, to implement such dependence using homogeneous models requires a division of the geographic region into multiple number of patches [15]."


2. In the previous review, I asked that the discussion address "application of this technique to areas of sparse spread/inflated zero counts" - I apologize if I was insufficiently clear.

In response, the authors discuss initial conditions, but the question was intended to be more about the application. In real epidemics, regions of low and varying population density often give rise to chaotic, rather than smooth, disease outcomes - virtually identical communities with the same level of ongoing spread observe vastly (on a proportional scale) different outcomes due to things like cluster outbreaks (long term care facilities, in the case of COVID) and super-spreader events. Unless I'm mistaken, the proposed deterministic technique could run into trouble when fit to actual data in such regions, particularly where the grid produces regions of sparse population. To be clear, I'm not suggesting that this limitation be addressed by the current work, but I think that it should probably be addressed in the discussion.

Stochastic evolution and chaotic behavior of the epidemic are very interesting issues, which will be added to the list of future studies. We do have in mind already how to handle stochastic behavior within our model. Indeed, it is out of the scope of this first paper that aims to introduce the model and show its main properties. As regard to adding a discussion on the limitations of our model in this respect, we feel that such a general discussion, without a deep analysis, will not be easily comprehended by the general readership, so we prefer to avoid it. We hope the referee can understand our viewpoint.

3. Myself and several reviewers noted the issue of long-distance transmission, and the authors added the language: "It should be noted that when normal life conditions are restored, our model has to be modified to include far-distance traveling; this can be readily done using long distance infections, which we defer to future work."

I don't think this sufficiently addresses the concern. Even in areas with strict lockdowns, people routinely travel greater distances than 1km, whether essential workers or performing essential tasks like grocery shopping. Coupled with the smooth nature of the deterministic framework, this seems like a very important limitation worthy of additional discussion.

As we mentioned, the parameter δ, that describe the node ("pixel") linear size, can be adjusted according to the effective mean distance traveled by people. Thus it can also be adjusted to 30 km or even greater. In fact, an alternative derivation of our model allows a variable range travel (e.g., exponentially decaying frequency) with a mean distance δ.

We do however understand the concern of the referee and thus added the following phrase to the first paragraph in the discussion section:

"… Nodes are defined by the assumption that within each node the frequency of contacts is still uniform, thus following a homogeneous model description. This allows to estimate the lateral (linear) size of a node δ (i.e. area equal to δ^2^, **for example by examining the mean distance traveled by people -- e.g., 30 km under moderate restrictions or normal life, or 1 km under strong lockdown --** and makes our model flexible enough to describe infectious disease spreading on a scale ranging from a neighborhood to a whole country."

4. When addressing the lack of uniform testing, the authors added the language: "Incomplete testing data is not going to severely hamper our predictions so long as it is uniform in space (e.g., only 10% of the COVID-19 positives are detected everywhere), and in general we may expect this to be so within a certain country where a uniform testing policy is adopted. Obviously, absolute predicted numbers will not be obtained without the use of an adjusting factor between tested and predicted numbers."

There are a couple of things which come to mind that may be worth addressing here as well:

a. For COVID specifically, testing was non-uniform over time essentially everywhere, due to the availability of tests. In many places spatial non-uniformity was/is observed as well.

We suppose the referee is correct about the spatial inhomogeneity of (the number of) tests. In fact, a similar challenge has been present also for the homogeneous models, as in most countries the number of tests changed considerably in the course of time. One good possibility, which has been used often so far, is to consider the fraction (percentage) of positive tests (from the number of tests performed) rather than their absolute number, and infer from them the relevant numbers. Another approach employed to obtain the unknown factor, is the comparison between post serological (antibody) test results and the qPCR test results, and for our purposes this requires local serological test results throughout the region. However, we believe it is not for us to solve all these fine technical issues when attempting to implement our model.

b. It's not clear to me that the lack of complete testing is a non-issue even if it is uniform - it seems that the dynamics on which the model is based perform very differently for different infectious fractions. It may be the case that the proposed model is sufficiently flexible to be fit to reported cases despite this limitation, but I'm not seeing that explicitly demonstrated in the manuscript. If I've missed something this would be helpful to clarify in the discussion section.

Indeed, the true values of sub-population fractions (of the total population number) is relevant for the predictions when the epidemic develops to the extent that the (local) susceptible population fraction, *h/n* in our notation,becomes significantly smaller than 1. When it is close to 1, the PDEs are essentially linear and one need not know the missing factor (between number of positive test and true number in the population) to predict the (relative) evolution. Again, clearly, homogeneous models also face the very same limitation, which can be partially overcome using practical approaches as discussed above (in the response to 4(a)); these are apparently performed already by different modeling groups that are part of (or working closely with) health authorities. We do not believe it is appropriate to dwell on such technical difficulties in this publication.

5. I also agree with the other reviewers that "population" and "sub-population" are confusing terms to use for disease states, since they conflict with the common practice of introducing strata into metapopulation style models (for example, age and geographic strata).

We do understand this possibility for confusion, however, the term "sub-population" is defined very clearly in the text. The other often used term "compartments" seems much more confusing to us, and the term "nodes" is already used to describe spatial nodes
